# Genome increase as a clock for the origin and evolution of life

**DOI:** 10.1186/1745-6150-1-17

**Published:** 2006-06-12

**Authors:** Alexei A Sharov

**Affiliations:** 1 Laboratory of Genetics, National Institute on Aging (NIA/NIH), 333 Cassell Dr., Baltimore, MD 21224, USA

## Abstract

**Background:**

The size of non-redundant functional genome can be an indicator of biological complexity of living organisms. Several positive feedback mechanisms including gene cooperation and duplication with subsequent specialization may result in the exponential growth of biological complexity in macro-evolution.

**Results:**

I propose a hypothesis that biological complexity increased exponentially during evolution. Regression of the logarithm of functional non-redundant genome size versus time of origin in major groups of organisms showed a 7.8-fold increase per 1 billion years, and hence the increase of complexity can be viewed as a clock of macro-evolution. A strong version of the exponential hypothesis is that the rate of complexity increase in early (pre-prokaryotic) evolution of life was at most the same (or even slower) than observed in the evolution of prokaryotes and eukaryotes.

**Conclusion:**

The increase of functional non-redundant genome size in macro-evolution was consistent with the exponential hypothesis. If the strong exponential hypothesis is true, then the origin of life should be dated 10 billion years ago. Thus, the possibility of panspermia as a source of life on earth should be discussed on equal basis with alternative hypotheses of de-novo life origin. Panspermia may be proven if bacteria similar to terrestrial ones are found on other planets or satellites in the solar system.

**Reviewers:**

This article was reviewed by Eugene V. Koonin, Chris Adami and Arcady Mushegian.

## Open peer review

Reviewed by Eugene V. Koonin, Chris Adami and Arcady Mushegian. For the full reviews, please go to the Reviewers' comments section.

## Introduction

The phenomenon of system complexity attracts attention of scientists from different areas of expertise from biologists and economists to mathematicians and cyberneticians [1,2]. Numerous approaches has been implemented to quantify complexity using estimates of probability, minimum length of encoding algorithm, etc. [1]. However little is known about the laws of evolution in complex systems. One of them is the Moore's law which states that the complexity and performance of certain systems associated with human technology (e.g., volume of published scientific information, number of nodes in computer networks, etc.) increases exponentially [3]. It is interesting if this law can be applied to other complex systems, e.g. to the large-scale evolution of living organisms.

Although the global increase of genome sizes from bacteria to mammals is a well-known fact [4], no attempt has been made to model this process. The total genome size appeared highly variable among organisms with the same level of morphological complexity, a phenomenon known as a C-value paradox [5]. These variations in genome size are caused mostly by gene duplication, polyploidy, and accumulation/deletion of intergenic DNA [6]. Thus, genome size was mostly studied as an indicator of insertion-deletion frequencies in different species [7] rather than a measure of complexity.

Biological complexity was recently defined by Adami et al. [8] as a size of functional and non-redundant genome. This measure does not depend on duplications, insertions, or deletions of non-functional or redundant sequences, and therefore it is more stable in evolution than the total genome size. The dynamics of genome increase in evolution can be modelled on the basis of known mechanisms which appear to act as positive feedbacks. First, the theory of a hypercycle considers a genome as a community of mutually beneficial (i.e., cross-catalytic) self-replicating elements [9]. For example, a gene that improves proof-reading increases the replication accuracy of all other genes. These benefits are applied not just to existing genes but also to genes that may appear in the future. Thus, already existing genes can help new genes to become established, and as a result, bigger genomes will grow faster than small ones. Second, new genes usually originate via duplication and recombination of already existing genes in the genome [4,10]. Thus, larger genomes provide more diverse initial material for the emergence of new genes. Third, large genomes support more diverse metabolic networks and morphological elements (at various scales from cell components to tissues and organs) than small genomes, which in turn may provide new functional niches for novel genes. These three mechanisms of positive feedback may be sufficient to cause an exponential growth in the size of functional non-redundant genome.

In some groups of organisms, the increase of genome size may be limited by organisational constraints. For example, prokaryotes have a small genome most likely because they had never developed mechanisms for preserving the integrity of a larger genome (e.g., better proofreading and mitosis). Lynch and Conery [11] showed that the effective population size, *N*_*e*_, is negatively related to the total genome size and number of genes, and explained this relationship by an increased genetic drift in small populations. However it is hard to agree with their interpretation of these facts that genome complexity "emerged passively in response to the long-term population-size reductions that accompanied increases in organism size". Positive selection may be equally important in the increase of the number of genes [12]. Thus, a better interpretation of the Lynch-Conery phenomenon is that large effective population size in prokaryotes was one of the constraints that slowed the increase of their genome size in evolution.

The exponential pattern of genome increase can be expected only in organisms that were most successful in overcoming organizational constraints. If all groups of organisms were equally successful in overcoming constraints, there would be no small genomes left. The only way to trace the increase of genome sizes is to compare groups of organisms that were most successful in overcoming organizational constraints, with most ancient groups, whose genomes did not increase due to constraints.

In this paper I address the problem of the rate of complexity increase in evolving living systems using Adami's [8] definition. Regression of the log functional genome size versus time of origin in major groups of organisms is consistent with the exponential hypothesis and indicates a 7.8-fold increase of complexity per 1 billion years. An interesting question is whether this rate of complexity increase can be extrapolated to the early (pre-prokaryotic) evolution of life, which I call a strong exponential hypothesis. If this strong hypothesis is true, then the origin of life should be dated ca. 10 billion years ago, i.e., before the formation of earth and solar system. I discuss potential arguments against the strong exponential hypothesis and show that they are not conclusive.

## Results

The size of functional non-redundant genome can be quantified using the Shannon's information measure [8], but currently this approach is not feasible because it would require unavailable information on fitness weight of each nucleotide in the genomic DNA. A simpler method is to consider functional genome as the size of coding and regulatory regions as they are known today.

**Figure 1:**
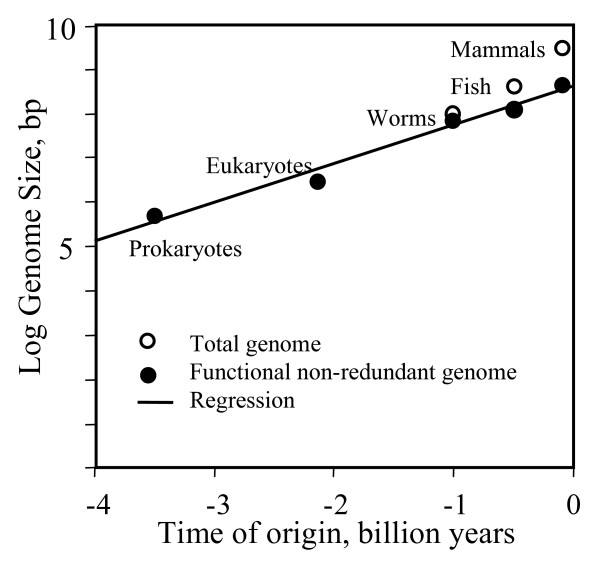
**Evolution of genome size on earth from prokaryotes to mammals**. Regression: log_10_(*y*) = 8.64 + 0.89·*x*, where *x *is time, billion yr, and *y *is the size of functional non-redundant genome, *R*^2 ^= 0.97.

The size of the functional and non-redundant fraction of genomes gradually increased in evolutionary time (Fig. 1). Mammals (mouse, rat, and human), which appeared just recently in earth history, have a genome of ca. 3.2 × 10^9 ^bp, however only 5% of it is conserved between species [13]. Conserved regions are definitely functional but there may be additional functional regulatory regions that are species-specific. These regions can be identified based on the absence of transposons, because transposons that are inserted in functional regions would interfere with normal gene regulation and eventually disappear due to natural selection [14]. Transposon-free regions of 5 and 10 kb account for 20%, and 12% genome size, respectively [14]. If we take 15% as a rough estimate, then the size of functional and non-redundant genome in mammals is ca. 4.8 × 10^8 ^bp. Fish existed 0.5 billion years ago [15]. The genome size of the fugu fish is 4 × 10^8 ^bp and 1/3 of it is occupied by gene loci [16]. Worms existed at least for 1 billion years [17]. The genome of the worm *Caenorhabditis elegans *has size of 9.7 × 10^7 ^bp and ca. 75% of its length is functional [18]. Eukaryote cells with mitochondria appeared between 2.3 and 1.8 billion years ago [19], and prokaryotes existed on earth as early as 3.5 billion years ago [20]. The date of eukaryote origin was estimated rather precisely (± 250 Mya) based on the homology of protein sequences [17]. Although there is abundant information on the size of genomes in contemporary prokaryotes and unicellular eukaryotes [21], most of it is not suitable for assessing the genome size of their early ancestors because the majority of these organisms had already increased their genome size since the origin of first prokaryotes and eukaryotes. Thus we were interested in the most primitive representatives of these groups. The smallest eukaryote genome (2.9 × 10^6 ^bp) was found in the microsporidia *Encephalitozoon cunicul *[22], and the smallest prokaryote genome size (5 × 10^5 ^bp) was found in *Nanoarchaeum equitans *[23] and *Mycoplasma genitalium *[24]. Prokaryotes and eukaryotes with the smallest genome are parasitic and may have a reduced genome size due to parasitism. However I selected them to get the most conservative estimate for the time elapsed since the origin of life. Also it is possible that the first prokaryotes and eukaryotes indeed had genome size comparable to contemporary parasitic species. Comparison of protein sequences indicates that the divergence time of archaebacteria, eubacteria, and eukaryotes occurred from 3.1 to 3.8 billion years ago [25].

I have not included plants into this graph for the following two reasons. First, their genomes are often highly redundant due to polyploidy [4], which makes it difficult to estimate the size of the functional non-redundant fraction. Second, functional non-redundant genomes in plants did not increase as fast as in vertebrates, and our goal was to trace the genomes in best performing groups of organisms. For example, the functional genome in *Arabidopsis thaliana *is ca. 3 times smaller and has more redundancy than in mammals [26], but flowering plants appeared simultaneously with mammals ca. 125 million years ago [27].

The increase of genome size approximately follows an exponential pattern (linear in the log scale) (Fig. 1). Because our estimates of genome size of first prokaryotic and eukaryotic cells are based on extrapolation rather than direct measurement, this regression cannot be viewed as a proof of the exponential hypothesis. We can only say that regression is consistent with this model and that the functional fraction of the genome increased approximately 7.8-fold per 1 billion years. Because two earliest points on the graph are most uncertain, we did a sensitivity analysis by varying these points within the limits of uncertainty (± 300 Mya, and ± 0.3 log bp). Then the rate of increase of functional genome changed in the range from 4.6 to 15.3 fold per 1 billion years.

The strong version of the exponential hypothesis is that the rate of genome increase can be extrapolated to the early (pre-prokaryotic) evolution of life. If this hypothesis is true, then the origin of life should be dated ca. 10 billion years ago, i.e. before the formation of earth and solar system, and implies panspermia (i.e., inter-stellar passive transport of living bacterial spores). Considering our sensitivity analysis, the date of life origin may vary from 7 to 13 billion years which is still greater than the age of earth.

## Discussion

We expect biological complexity measured by the size of functional non-redundant genome to increase exponentially with time because of several positive feedback mechanisms, including well-studied phenomena of gene cooperation and duplication [9,10]. Available data on genome size of major groups of organisms from prokaryotes to mammals is consistent with the exponential hypothesis (Fig. 1) although it not sufficient to prove it. Thus, the Moore's law of exponential growth may be true not just in the area of human technology but also in the macro-evolution of living organisms. According to our regression, the size of functional non-redundant genome of living organisms on earth increased approximately 7.8-fold per 1 billion years.

The idea of using the volume of genetic information as a measure of biological complexity has been developed in studies of "digital organisms" in a computer medium [8]. The genome of these organisms determined their strategy of survival and replication in a partially predictable environment. Natural selection in information-rich simulated environments lead to the increase of genome size as digital organisms progressively accumulated and encoded the information about their environment, whereas selection in information-poor environments lead to the decrease of genome size because there was no new information to encode and the old information became better compressed [28]. However, evolution of digital organisms was too short to observe acceleration effects of positive feedback mechanisms that may lead to an exponential increase of genome size.

In our model we assumed a uniform rate of exponential increase in biological complexity in most successful taxonomic groups. But other lineages have lower rates of complexity increase. For example, in *Archaea *and *Eubacteria*, the genome size increased only 1.9 and 2.5 fold per 1 billion years, respectively (these estimates are based on the largest known archaeal genome, 5 Mb, in *Methanogenium frigidum *and bacterial genome, 13 Mb, in *Sorangium cellulosum *[21]). The difference between the rates of increase of genome complexity between most successful and lagging lineages can be explained by evolutionary constraints of the latter ones (e.g., inefficient DNA proofreading and absence of mitosis). Another possibility is that prokaryotes always had slower rates of complexity increase than eukaryotes, for example, due to their larger effective population size [11]. If this is true, then the rate of the "complexity clock" increased with the emergence of eukaryotes, and therefore, life may have originated even earlier than expected from the regression in Fig.1.

The fundamental question is what the rate of complexity increase was in early (pre-prokaryotic) evolution compared to the rate observed in Fig. 1. According to available data, the exponential rate of increase of genome complexity in primitive organisms is not higher than in evolutionary advanced organisms, and even tend to be smaller (see above). Thus, I propose a strong exponential hypothesis that rates of increase in genome complexity in early evolution did not exceed those in later evolution. If this hypothesis is true, then life originated long before the development of the solar system (ca. 10 billion years ago or even earlier), and we have to assume that early earth was contaminated with bacterial spores from some other stellar system (i.e., panspermia). Possible objections to this strong exponential hypothesis are: (1) there is a threshold of genome complexity that can support life and adaptive evolution of organisms, hence life started from an accidental emergence of a relatively complex genome; (2) even if genome size increased gradually, this process was much faster in early evolution than after the emergence of prokaryotes [29]; and (3) the exponential hypothesis should be rejected because panspermia seems highly unlikely [30].

The idea of a minimum complexity of self-reproducing systems stems from Von Neumann's theory [31]. He argued that self-reproducing systems require a universal constructor, defined as an algorithm for a Turing machine that can construct any system (e.g., within a 2-dimensional cellular automata) based on its linear description. The complexity of universal constructors cannot fall below some minimum level which is rather high (ca. 10^5 ^cells with 29 states each). However Von Neumann's model of self-reproduction is too stringent and there is little evidence that living organisms contain universal computation devices [32]. Cellular automata models showed that systems don't need to be complex to self-reproduce and even evolve [33]. It may be argued that evolution of simple systems is always limited, whereas the universal constructor guarantees an unlimited "open-ended" evolutionary potential. But evolution can progress even if it has limitations. Eventually some limitations are removed and evolution goes further. Thus, there is no need to assume unlimited evolutionary potential from the very beginning. First living systems had much simpler interpretation machinery, than contemporary cells; thus, most of their mutations were lethal. Because of this, the rates of evolution and complexity increase were low in agreement with the exponential model. As the complexity of living systems increased, more genetic variation became inheritable and the growth of complexity accelerated. Eventually, the interpretation of genomic information became closer to universal, but still it has not reached the state of full universality.

There are several models of ancient living cells that preceded prokaryotes. The "RNA-world" model assumed that RNA was used both for catalysis of various chemical reactions and for information transfer between generations [9,34,35]. The RNA-world cell required multiple genes including RNA-polymerase, catalysts for synthesis of sugars, nucleotides and phospholipids, and for energy storage and processing. Spontaneous self-assembly of such a complex system had an extremely low probability even if all components were immediately available and brought together (e.g., *p *= 10^-126 ^for 400 bp DNA). But the probability that all components were simultaneously available in a very small volume is much lower than the probability of assembly. Even a single nucleotide is too complex to appear spontaneously. Both heterocyclic bases and sugars are rare chemicals in unanimated world, and the probability of their reaction is negligible. Accumulation of multiple nucleotides is near to impossible. Although the probability of self-assembly is greater than zero, the expectation time may appear longer than the age of the universe. One of the major features of evolution is its continuity when even large changes occur via accumulation of small intermediate steps. Evolution is continuous because a large quantum leap requires a longer expectation time than a series of smaller changes. Thus, if one of competing lineages implements small changes, and the other waits for a favourable big change, the first one eventually wins.

Alternative models assume that first self-reproducing systems were very simple and had only a few bits of inherited information which were transferred to offspring as a multi-component mixture [36-38]. Later coding elements became organized into sequences, which were very short and noisy at the beginning, and then increased their stability and length. The main difference of this approach from the "RNA-world" is that coding elements were not descriptions (icons) of system elements but simple indexes. The genome of contemporary cells indeed describes the sequence of proteins, but there are many cell components that have no description in the genome. For example, the structure of nucleotides, aminoacids, sugars, steroids, and other small molecules is not coded in the DNA sequence. However cells manage to produce and operate these molecules by using indexes, e.g. proteins that bind and transform these molecules. Indexes can be much simpler than descriptions because they do not contain all information about objects to which they point. If a molecule in addition to auto-catalysis (equivalent to DNA replication) can support directly or indirectly an additional process which is beneficial for the whole cell, then it can be viewed as an element that encodes this process. These models of biogenesis via evolution of very simple self-reproducing systems are more realistic than the assumption of self-assembly of an "RNA-world" cell.

Koonin and Galperin [29] proposed that rates of evolution may have been much faster immediately after the origin of life than in subsequent history of pro- and eukaryotes because it was driven mostly by positive selection in the absence of competition. As competition increased, positive selection was largely replaced by purifying selection which simply preserved the optimum structure and function of organisms and resulted in slower evolutionary rates. This hypothesis is closely linked to the theory of punctuated equilibrium [39] which assumes that evolutionary rates are highly unstable and long periods of evolutionary stagnation are occasionally interrupted by short periods of very rapid change of flora and/or fauna caused by either external impact or loss of ecosystem stability. The main support for this hypothesis comes from paleontology which clearly shows rapid changes in the composition of major taxonomic groups during very limited time.

Positive and purifying forms of natural selection indeed alter in time and space, and purifying selection is more dominant in favourable and stable habitats. However it is wrong to see evolutionary novelty only in positive selection. According to Schmalhausen [40], who introduced these two forms of selection, purifying selection may be even more innovative than positive selection. Positive selection is guided by immediate selective advantage, whereas purifying selection may optimise the function of already existing adaptations (e.g., reduce energy costs, increase reliability and adaptability). Thus, there is no reason to expect that the rate of increase in biological complexity under purifying selection was lower than under positive selection. Another argument is that rapid changes in flora and fauna observed in fossil records do not necessary represent the emergence of novel adaptations. It is known that predecessors of at least some large taxonomic groups appeared long before the time of their massive expansion. Thus it is possible that the increase of biological complexity was rather uniform in time but the numerical expansion of certain taxonomic groups occurred rapidly within short periods.

Koonin and Galperin [29] considered viruses as an example of rapid evolution which may resemble the pattern of evolution in pre-prokaryotes. I think this comparison is questionable because viruses are parasites and their evolution is not subject to constraints of a free-living organism. Viruses do not have to spend time and resources on mechanisms of transcription, translation, and homeostasis; instead they simply destroy the host cell. The accuracy of their replication is low; thus they would get extinct due to the error catastrophe [9] if they were free-living and had to keep all necessary genes. Also, rapid evolution does not imply the growth in complexity, and there is no evidence of high rates of complexity increase in viruses. Thus, neither the punctuated equilibrium theory nor the virus example support the idea that early evolution of life on earth was much faster than the subsequent evolution from prokaryotes to mammals.

The majority of experts in the area of biogenesis reject the hypothesis of panspermia [41], possibly because it seems to leave little chances to decipher the process of biogenesis. However, clues of biogenesis can be found in cellular metabolism [42], no matter on what planet did life originate. Experimental approaches to the study of the origin of life also do not depend on the time and place of biogenesis. Recent analysis indicated the possibility of inter-stellar transfer of microbial biota [43]. Contaminated material can be ejected into space from a planet via collision with comets or asteroids [44]. Then bacterial spores may remain alive in a deep frozen state for a long time that may be sufficient for inter-stellar transfer. One of the scenarios of life arrival to new planets is the capture of small contaminated particles by a protoplanetary disc before planet formation [43]. Panspermia is a testable hypothesis; it may be proven if living bacteria are found on any planet or satellite in the solar system other than earth, and if these bacteria have the same nucleic acids (RNA or DNA) and similar mechanisms of transcription and translation as in terrestrial bacteria.

All known living organisms have only slight variations in the coding mechanism and enzymes involved in DNA replication, whereas fundamental features including complimentary mechanism of DNA replication and transcription, and codon-based translation are universal, which implies a common ancestor with a genome of ca. 300 genes for supporting these processes [45]. Life on earth became possible ca. 4 billion years ago, and the earliest fossil records of living organisms similar to modern bacteria are dated 3.5–3.8 billion years ago [20,41]. Thus, very little time (a few hundred million years) was left for the origin of life if it happened on earth. In this case we have to assume that the functional genome size increased by 5 orders of magnitude during the first few hundred million years, and then biological evolution suddenly slowed down so that the genome size increased only 3 orders of magnitude during the next 3.5 billion years. This could happen only in the case of strict constraints on the increase of genome size, which contradicts to the C-value paradox. The panspermia hypothesis lifts this major time constraint which I believe will bring more realism to future theoretical and experimental studies of biogenesis. It should be discussed on equal basis with alternative hypotheses of de-novo life origin on earth.

## Reviewers' comments

### Reviewer's report 1

#### Eugene V. Koonin, National Center for Biotechnology Information, NIH, Bethesda Maryland, USA

What determines the total size of genomes and their effective complexity (sensu Adami) and how did genome size evolve throughout life's evolution are genuinely exciting and fundamental biological issues. Potentially, a lot of information can be extracted from comparative analysis of genome size and complexity. This paper is an attempt to cast this analysis in the simplest possible terms, i.e., to back-extrapolate the maximum genome size attained on earth at different times (I believe this is what is being used to produce the plot in Fig. 1; the corresponding language in the paper is not very precise) to the origin of the first organisms. The inferred dates for the origin of life are very early and, under a straightforward interpretation favored by the author, suggest that life did not begin on earth but rather elsewhere in the Universe some 10 billion years ago, after which it spread by panspermia.

I am not at all a priori prejudiced against the panspermia hypothesis and actually agree with the author's concluding sentence in that panspermia should be considered "on equal basis with alternative hypotheses of de-novo life origin on earth". However, I think that the approach used in this work provides no support for an early date of life's origin. The main problem, as I see it, lies with the fact that the key plot in Fig. 1 combines two worlds with very different evolutionary trends, the prokaryotes and the eukaryotes (especially, complex, multicellular eukaryotes). The exponential law very well might hold for the portion of the curve that corresponds to complex eukaryotes (or, possibly, eukaryotes in general), and the reasons why this is so would be interesting to discuss in some depth (more data points would be required, though). The problem is, however, that, for the first 1.5–2 billion years of life's evolution on this planet, all existing life forms were prokaryotes. There is just one point corresponding to prokaryotes in Fig. 1, and there is, indeed, an excellent reason for that: we have no evidence whatsoever that the maximum genome size of prokaryotes increased during that enormous time span or in the time elapsed since.

##### Author's reply (1)

I have addressed this problem in discussion by estimating the average rate of increase in genome complexity in Archaea and Eubacteria which appear lower than the rate of complexity increase in eukaryotes. Then I discuss 2 possible scenarios: (a) initial rates of complexity increase in prokaryotes were similar to those observed in eukaryotes and then slowed down due to organization constraints, or (b) rates of complexity increase in prokaryotes were always slower than in eukaryotes. With scenario (a), the expected origin of life is ca. 10 billion years ago according to regression (Fig. 1), and with scenario (b), life originated even earlier than that. Thus, separate handling of prokaryotes and eukaryotes does not bring the predicted date of life origin closer to present.

For all we know, the characteristic complexity of the prokaryotic genomes had been reached very early on during life's evolution (considering the geochemical and paleontological evidence of more or less modern-like microbiota ~3.5 billion years ago) and remained in equilibrium ever since. Thus, to the best of our understanding, there was an early explosive phase of evolution of complexity, which was followed by stasis (the prokaryotic phase of life's history) and then by another burst associated with eukaryogenesis. The authors dismisses, very lightly, the notion of punctuated equilibrium. This is not the place to assess the validity of the specific theory of Gould and Eldredge (it might indeed have its problems), however, I believe that, in general, major non-uniformity of the tempo of life's evolution cannot be denied.

##### Author's reply (2)

If the rate of evolution is measured by numerical expansion of some taxonomic groups and numerical decline of other groups, then it is definitely non-uniform. However, in the paper I discuss the rate of increase in genome complexity which is an entirely different process. So far there is no evidence that the rate of complexity increase fluctuated considerably over time. In particular, there is no evidence of "early explosive phase of evolution" of prokaryotes and "another burst associated with eukaryogenesis". Genome complexity can increase even if direct adaptations to the environment remain stable (due to increasing reliability, modularity, and adaptability).

In the general epistemological sense, the approach to back-extrapolation of life's history taken in this paper can be characterized as ultra-uniformitarianism, a wordlview championed by the great geologists Hutton and Lyell and strongly embraced by Darwin (this work even might be considered something of an extension of this view but the spirit is definitely the same). In that vein, I believe that what is done here is an interesting exercise because it showcases the kind of conclusions to which ultra-uniformitarianism can lead. If the entire discussion and conclusions were rewritten along these lines, this could turn into a sound piece.

There are two issues in this paper that are not as germane to its main conclusions as the above but are important and deserve comment because they are not, I believe, adequately addressed. The first issues is the nature on constraints that effect evolution of genome complexity/size. The authors dismisses Lynch and Conery's population-genetic concept of genome complexity evolution (his ref. [12]) by citing the comment of Charlesworth and Barton [13]. This is, I think, disingenious because Charlesworth and Barton's note (regardless of whether or not their arguments are compelling) does not even seek to invalidate Lynch's theory as a whole but rather addresses specific issues of mobile element propagation. I strongly believe that Lynch's concept has a lot going for it and explains an important, if not the central, aspect of these constraints.

##### Author's reply (3)

I have removed most of my criticism of Lynch and Conery paper because I agree that their data are valid. However I disagree with their evolutionary interpretation, and suggest another interpretation that large *N*_*e *_was one of the constraint in the evolution of prokaryotes.

Another, complementary source of these constraints that is not at all covered is the faster than linear scaling of the number of regulatory genes with genome size (van Nimwegen E. Trends Genet. 2003 Sep;19(9):479–84; Konstantinidis KT, Tiedje JM. Proc Natl Acad Sci U S A. 2004 Mar 2;101(9):3160–5).

##### Author's reply (4)

I agree that the proportion of regulatory genes may change in evolution. However I don't think that this can substantially affect the regression line which I discuss in the paper.

Another issue is that of the "minimal genome": equating minimal genomes reconstructed by comparative-genomic approaches with ancestral life forms is incorrect and does not reflect the original view of the authors of the minimal genome notion (of which ref. 27 in the present manuscript is a proper reflection).

##### Author's reply (5)

I have removed the reference to the "minimal genome" paper in the paragraphs where I discuss the complexity of ancestral life forms and the possibility of spontaneous self-assembly of complex systems.

Again, all this is not to claim, with confidence, that the only form of life we are aware of evolved on earth rather than elsewhere in the universe. The latter is quite a possibility. The only claim I am making is that the data analyzed in this paper and, for that matter, any comparative-genomic data I can think of do not provide any evidence in support of an early, extraterrestrial origin of life. Accordingly, I believe that terrestrial origin around 4 billion years ago should be taken as the null hypothesis.

##### Author's reply (6)

I do not claim to have a proof for the exponential hypothesis, but offer available supporting evidence. In addition, I suggest (a) mechanisms of positive feedback that can cause the exponential increase in genome complexity and (b) possible test for panspermia if life is found on any planets or satellites in the solar system. Testing multiple null hypotheses may appear more productive than testing a single one.

### Reviewer's report 2

#### Chris Adami, Keck Graduate Institute, California Institute of Technology, Pasadena, USA

In this contribution, the author attempts to characterize the functional form of the relationship between the sizes of the functional genome of organisms and their appearance in the fossil record. Using five data points (prokaryotes, eukaryotes, worms, fish, and mammals), the author deduces an exponential increase in functional size with time. He then uses this functional relationship to hypothesize an origin of life that exceeds the age of the Earth by a factor of two. From this he concludes that the origin of life cannot have taken place on Earth, but points towards hypotheses of the panspermia type.

This paper is an example of how not to analyze data. First, there is no doubt that a much more sophisticated analysis of whole genome data can be performed. For example, the author claims that 1/3 of the Fugu rubripes genome is functional (this is one of his datapoints), but the original publication only states that "gene loci occupy about one-third of the genome". There is some evidence that non-coding but functional (likely regulatory) DNA increases with the complexity of the organism (see, e.g., [1]), so that taking just the gene loci into account is very likely to be misleading, more so for complex metazoans.

##### Author's reply (7)

I believe that my estimate of functional genome size of *Fugu rubripes *as 1/3 of genome is realistic. Gene loci contain more than coding sequence; they also include introns and untranslated regions. Although I did not explicitly include promoter sequences, they may be of similar size as non-functional portion of introns. This analysis is not sensitive to small variation in functional non-redundant genome size (± 20–30%). This level of uncertainty is inevitable because we do not have an exact quantitative measure on genome complexity.

Even were we to accept the five data points at face value, they would not allow us to reach any conclusion about the origin of life. This is a classical case of "allowing the data to suggest a model". For example, I have a time series of personal Marathon finishing times versus date that very much suggests a linear (decreasing) relationship (with four, rather than five, data points). But I am not so foolish as to predict from these data points the date when I will break the world record (or the speed of sound, or light, for that matter). The authors advance some arguments for their exponential model, but many more arguments speak against it. For example, while an approximately exponential growth could be argued for in any particular period, major changes in organization (for example from unicellular to multicellular) are likely to affect the rate of growth, so that a piecewise exponential would be a more reasonable assumption.

##### Author's reply (8)

see reply #1 to Eugene Koonin

Even more dramatic, it is inconceivable that life began with just a few nucleotides. Instead, there must have been an initial step–from zero to finite–in the complexity of organisms (as measured by its functional genome). The size of this step will then be crucial in determining the point of origin.

##### Author's reply (9)

I have added more discussion on why it is more likely that genome evolved gradually from single coding elements (paragraphs 5–7 of Discussion).

But as we have no information about the minimal genome size of living organisms, an extrapolation with a pure exponential simply makes no sense. Thus, while a thorough analysis of the evolution of functional genome size would certainly be welcome, the data presented here do not warrant any conclusion, except perhaps that the size of functional DNA has been increasing in evolution, something we should not be terribly surprised to learn.

### Reviewer's report 3

#### Arcady Mushegian, Stowers Institute, Kansas City, USA

I agree with the Author on the following:

1. If there is evidence supporting panspermy, it should be considered seriously.

2. Panspermy, if it occurred, should not prevent us from attempting to reconstruct ancestral genomes, using comparative genomics and the knowledge of planetary chemistry.

3. Early stages of evolution of Life seem to have been overloaded with evolutionary innovation, which asks for explanations. Panspermy may be one such explanation; periods of accelerated evolution, prompted in part by Lynch-Conery considerations of Ne, is another.

Having said that, I do not see any striking arguments for panspermy in this work. The "genome size as a clock" approach is, in my opinion, qualitatively correct, and it shows what we already knew, i.e., that the earliest stages of life appear to have had precious little time to progress to what are currently our best estimates of genome size and the number of protein-coding genes (on the latter, see also below). Whether the dependency is of the exponential form, however, remains to be seen.

##### Author's reply (10)

see reply #6 to Eugene Koonin

Discussion of minimal genome in this regard is a red herring. First, the Author misreads what is in the minimal-genome literature (e.g. Mushegian and Koonin, 1997; later reviews both by myself and by Koonin; and experimental work of Hutchison, Smith and Venter, most recently Glass et al., 2006; Pubmed 16407165). Minimal genome is a construct of biochemical engineering, predicted or directly manipulated to sustain life in a rich medium with the smallest number of genes. It is not purported to model the ancestor, even though it, same as the ancestral genomes, may be constructed using methods of comparative genomics, and even though minimal genome may be enriched in ancestral genes. Second, no one ever said that the minimal or ancestral genomes have evolved by spurious assembly of 300 genes – any paper, including our own, that speculates about origins of Life, understands the problem of earlier stages clearly.

##### Author's reply (11)

see reply #5 to Eugene Koonin

Third and most important, all this is not relevant to Author's own argument: the genome to discuss is not minimal one, but that of LUCA (last universal common ancestor). The latest reconstructions of LUCA gene content, notably Pubmed 12515582 and 16431085, come up with 600–1000 genes, which is in fact even better for the early-overload argument, so why not stick to these estimates?

##### Author's reply (12)

In this paper I used existing genomes, and LUCA is only mentioned for discussion purposes. Also I tried to make my estimates for predicted life origin as conservative as possible.

Ultimately, the question is not whether "early genomes were way too complex", but, in the likely case that they were, whether panspermy better explains these observations than other hypotheses. I find it counterproductive to dismiss the Lynch-Conery theory in one sentence – at least in the sentence that directs to the Charlesworth-Barton paper, as if it is the last word on the subject. In fact, said paper is rather supportive of many observations and explanations presented by Lynch and Conery, arguing mostly with the idea of subfunctionalization (where Charlesworth's argument is an overly general one, which is understandable: coming up with any specifics here will require a lot of quite subtle analysis of the data that are not there yet) and, in a technically involved way, with the ideas of transposon dynamics (which, I think, are addressed in part by M.Lynch in Pubmed 16280547). If the author has a substantive disagreement with Lynch-Conery, let us hear it, but we haven't yet.

##### Author's reply (13)

see reply #3 to Eugene Koonin

The "viral hypothesis", in the meantime, exists in many modifications, not all of which require modern-type viruses: see for example, Woese (series of essays in 1998–2002) and Koonin-Martin (Pubmed 16223546). With regards to absolute time scale, however, these theories may not be even that helpful, because the step from these general hypotheses to constant vs variable evolutionary rate would not be trivial.

##### Author's reply (14)

Even if early viruses were different (e.g., non-parasitic) there is no evidence that their rate of complexity increase was higher than in eukaryotes.
